# Characterization of a *Bacillus velezensis* strain isolated from *Bolbostemmatis Rhizoma* displaying strong antagonistic activities against a variety of rice pathogens

**DOI:** 10.3389/fmicb.2022.983781

**Published:** 2022-09-28

**Authors:** Jianping Zhou, Yunqiao Xie, Yuhong Liao, Xinyang Li, Yiming Li, Shuping Li, Xiuguo Ma, Shimin Lei, Fei Lin, Wei Jiang, Yong-Qiang He

**Affiliations:** ^1^National Demonstration Center for Experimental Plant Science Education, College of Agriculture, Guangxi University, Nanning, Guangxi, China; ^2^State Key Laboratory for Conservation and Utilization of Subtropical Agro-Bioresource and College of Life Science and Technology, Nanning, China; ^3^State Key Laboratory for Conservation and Utilization of Subtropical Agro-Bioresources, South China Agricultural University, Guangzhou, China; ^4^Key Laboratory of Natural Pesticide and Chemical Biology, Ministry of Education, South China Agricultural University, Guangzhou, China

**Keywords:** *Bacillus velezensis*, *Bolbostemmatis Rhizoma*, biological control, broad-spectrum antagonistic activities, rice pathogens

## Abstract

Biological control is an effective measure in the green control of rice diseases. To search for biocontrol agents with broad-spectrum and high efficiency against rice diseases, in this study, a strain of antagonistic bacterium BR-01 with strong inhibitory effect against various rice diseases was isolated from *Bolbostemmatis Rhizoma* by plate confrontation method. The strain was identified as *Bacillus velezensis* by morphological observation, physiological and biochemical identification, and molecular characterization by 16S rDNA and *gyrB* gene sequencing analysis. The confrontation test (dual culture) and Oxford cup assays demonstrated that *B. velezensis* BR-01 had strong antagonistic effects on *Magnaporthe oryzae*, *Ustilaginoidea virens*, *Fusarium fujikuroi*, *Xanthomonas oryzae* pv. *Oryzicola*, and *Xanthomonas oryzae* pv. *oryzae*, the major rice pathogens. The genes encoding antimicrobial peptides (*ituA*, *ituD*, *bmyB*, *bmyC*, *srfAA*, *fenB*, *fenD*, *bacA*, and *bacD*) were found in *B. velezensis* BR-01 by PCR amplification with specific primers. *B. velezensis* BR-01 could produce protease, cellulase, β-1,3-glucanase, chitinase, indoleacetic acid, siderophore, and 1-aminocyclopropane-1-carboxylate (ACC) deaminase, and might produce three lipopeptide antibiotics, surfactin, iturin, and fengycin based on Liquid chromatography–mass spectrometry (LC-MS) results. Furthermore, the plant assays showed that *B. velezensis* BR-01 had significant control effects on rice bacterial blight and bacterial leaf streak by pot experiments in greenhouse. In conclusion, *B. velezensis* BR-01 is a broad-spectrum antagonistic bacterium and has the potential as the ideal biocontrol agent in controlling multiple rice diseases with high efficiency.

## Introduction

Rice (*Oryza sativa* L.) is one of the most important staple crops, which is widely cultivated around the world and mainly in Asia. It is also one of the earliest domesticated crop species. Due to the continuously human intervening, rice plants might be more fragile to stresses than many other crops, and more susceptible at different seasons and their growth stages (Ou et al., 2014; Zhao et al., 2014; Quibod et al., 2020). Rice diseases have caused great losses in rice production, both in yield and quality. Chemical control has played a major role in rice disease control with the characteristics of convenience, rapidity, and the remarkable effects. However, the non-negligible side effects of many chemical agents, such as chemical residues, environmental pollution, and pest resistances, are clearly inconsistent with the requirements in sustainable agriculture (Jepson et al., 2020). The selection and application of disease-resistant varieties is the preferential choice for green agriculture, but the long breeding cycles and the uncertain resistant persistency of the resistant cultivars are still major challenges in rice production (Deng et al., 2020). Biological control, a sustainable and practical approach for plant disease management, is a promising alternative strategy in rice disease prevention and control (Ab Rahman et al., 2018). So far, a variety of microorganisms, mainly including *Bacillus*, *Pantoea*, *Streptomyces*, *Trichoderma*, *Clonostachys*, *Pseudomonas*, *Burkholderia*, *Lysobacter*, and yeasts, have been shown to have the antagonistic ability against rice pathogens and some of which have been registered as the biopesticides used in controlling rice diseases in fields (Qian et al., 2009; Limtong et al., 2020; Rani et al., 2021; Doni et al., 2022; Tan, 2022; Zhang H. J. et al., 2022).

Among the most commonly used antagonistic microorganisms, *Bacillus* is a prominent genus used as the biocontrol agents in rice production, with strong stress resistance and good environmental adaptability (Doni et al., 2022). *Bacillus* is a large and heterogeneous collection of aerobic or facultatively anaerobic, rod-shaped, endospore-forming bacteria that are widely distributed throughout the environment (Rabbee and Baek, 2020). Currently, *B*. *subtilis*, *B*. *cereus*, *B. pumilus*, *B*. *amyloliquefaciens*, *B. firmus*, *B. megaterium*, *B. safensis*, and *B. velezensis*, etc., have been reported to have the antagonist effects on plant pathogens, including bacteria, fungi, and nematodes (Lopes et al., 2018; Chakraborty et al., 2021). Numerous studies have shown that different taxa of *Bacillus*, even different strains of the same species in *Bacillus*, might have the distinct biological functions on plant pathogens. Genomic analysis has revealed that *Bacillus* spp. possess species- or strain-specific clusters of genes related to the biosynthesis of secondary metabolites, which play different roles in both pathogen suppression and plant growth promotion (Rabbee and Baek, 2020; Doni et al., 2022). The biological control mechanisms of *Bacillus* are mainly described as the production of direct antifungal substances such as volatile organic compounds (VOCs), non-volatile metabolites, and extracellular lytic enzymes. *B*. *subtilis* is the well-studied antagonist organism group in controlling rice blast, sheath blight, and bacterial leaf blight, which can produce mainly bacteriocin and lipopeptide compounds, organic acids, and chitinases to suppress pathogen either directly or through enhancing the plant defense mechanisms (Amruta et al., 2016; Kaspar et al., 2019; Doni et al., 2022). In addition to *B*. *subtilis*, other *Bacillus* spp. were also demonstrated to exert effectively antagonistic on rice pathogens (Chakraborty et al., 2021). Yu et al. (2002) found that production of iturin A by *B. amyloliquefaciens* suppressing *Rhizoctonia solani*. Saechow et al. (2018) isolated a strain of *B. amyloliquefaciens* BAS23 from rice field soil. Based on dual culture method results, BAS23 showed strong inhibitory activity against a broad range of dirty panicle fungal pathogens of rice. It also has a variety of characters to promote plant growth and improve the growth of rice seedlings in the test tube. Jain et al. (2020) demonstrated that synergistic consortium of beneficial microbes *B. amyloliquefaciens* and *Aspergillus spinulosporus* in rice rhizosphere promoted host defense *Xanthomonas oryzae* pv. *oryzae*. *B. cereus*, *B. firmus*, *B. megaterium*, *B. pumilus*, and *B. safensis*, etc., have also positively evaluated having multiple functions in biocontrol of rice diseases (Kanjanamaneesathian et al., 2009; Rong et al., 2020; Sha et al., 2020; Chakraborty et al., 2021).

*Bacillus velezensis* is a newly reclassified *Bacillus* species, which consists of the original *B*. *velezensis* strains (Ruiz-García et al., 2005) and *B. amyloliquefaciens* subsp. *plantarum*, *B. methylotrophicus*, and *B. oryzicola*, also known as later heterotypic synonyms of *B*. *velezensis*, based on phylogenomics (Dunlap et al., 2016; Fan et al., 2017; Adeniji et al., 2019). Studies showed that many strains of this species have the ability to suppress the growth of rice pathogens and promote plant growth, largely relying on the gene clusters related to the biosynthesis of secondary metabolites, e.g., the cyclic lipopeptides (i.e., surfactin, bacillomycin-D, fengycin, and bacillibactin) and polyketides (i.e., macrolactin, bacillaene, and difficidin) (Rabbee and Baek, 2020). At present, hundreds of *B. velezensis* strains have been isolated from different environments, some of which were demonstrated to have antagonistic ability to rice pathogens in laboratories or/and in the fields. *B. velezensis* (synonyms *B. methylotrophicus*) strain BC79, isolated from primeval forest soil in Qinling Mountains, China, was able to suppress mycelial growth and conidial germination of *M. oryzae* in dual cultures on solid media, mainly by the phenaminomethylacetic acid (Shan et al., 2013). *B*. *velezensis* (formerly *B*. *amyloliquefaciens*) FZB42, the model strain for Gram-positive plant-growth-promoting and biocontrol rhizobacteria, was shown to possess biocontrol activity against *X. oryzae* pv. *oryzae* and *X. oryzae* pv. *oryzicola* by producing the antibiotic compounds difficidin and bacilysin (Wu et al., 2015; Fan et al., 2018). *B. velezensis* (synonyms *B. oryzicol*a) strain YC7007, an endophytic bacterium isolated from the roots of rice, could inhibit the growth of important fungal and bacterial pathogens of rice, such as *Fusarium fujikuroi* and *Burkholderia glumae via* antibiotic production and systemic resistance inducing activities (Chung et al., 2015; Hossain et al., 2016). *B. velezensis* G341, isolated from Korean ginseng root with rot symptoms, could inhibit mycelial growth of *M. oryzae* and *R. solani*. It was found that the diffusible and volatile antifungal compounds emitted from strain G341 were found to have strong effects on *R. solani* than *M. oryzae* (Lim et al., 2017). *B. velezensis* NKG-2 could inhibit the growth of *U. virens* on potato glucose broth (PDB), which had the ability to produce chitinase, cellulase, β-1,3-glucanase, amylase, indole-3-acetic acid (IAA), and siderophore (Myo et al., 2019). *B. velezensis* E69, isolated from rice leaf, could strongly inhibit the conidial germination and appressorial formation of *M. oryzae* (Sha et al., 2019). *B. velezensis* XT1, a halotolerant bacterium isolated from a saline habitat in Spain, had antifungal activity *in vitro* by inhibiting the mycelial growth of *M. oryzae* and promoting the growth of many plants (Torres et al., 2019). A marine-derived strain *B. velezensis* 11-5 could produce cyclic lipopeptide iturin A which could inhinbit *M. oryzae* growth (Ma et al., 2020). C_15_surfactin A, produced by *B. velezensis* HN-2, displayed antibacterial activity against *Xoo* and effectively inhibited its infection of rice (Jin et al., 2020). *B. velezensis* CMRP 4490 had antagonistic activity against *R. solani* (Teixeira et al., 2021). *B. velezensis* JK23, a radiation-resistant strain isolated from radioactive contaminated soil, had strong inhibition against *F. moniliforme* (Zhang et al., 2021). *B. velezensis* ZW10, isolated from Sichuan basin neutral purplish soil, was reported to have strong antagonistic on *M. oryzae* growth on plate and the capacity to stimulate rice defenses (Chen et al., 2021). *B. velezensis* J17-4, isolated from healthy and sterilized stems of rice stem, could significantly inhibit the growth of *Dickeya zeae* and could significantly reduce the inhibitory effect of *D*. *zeae* strain EC1 against rice seed germination under laboratory inoculation conditions (Shi et al., 2022). These researches indicated that *B. velezensis* has strong antagonistic effects on a variety of rice diseases, from numerous individual cases and a few of *B. velezensis* strains had already been registered as biopesticides playing important roles in rice disease prevention and control (Doni et al., 2022; Zhang Y. H. et al., 2022). However, the studies also showed that *B. velezensis* strains seemingly differ in their abilities to control certain rice diseases, which might be insufficient to meet the requirements of high efficiency and low-carbon in green rice production. The broad-spectrum *B. velezensis* strains with versatility in antagonistic, host immunity inducing, and plant-growth-promoting, are urgently needed to protect paddies from the attacks of an armada of diseases simultaneously (Shasmita et al., 2022).

In recent years, endophytic microorganisms from medicinal plants and their active components have shown good development value in agriculture, fermentation industry and pharmaceutical industry (Zhao et al., 2011; Gond et al., 2012; Kuo et al., 2021; Zhang G. Y. et al., 2022). However, these studies mainly focus on endophytic fungi. In this study, a broad-spectrum biocontrol bacterium, *B. velezensis* BR-01, was identified from *Bolbostemmatis Rhizoma*, a Chinese herbal medicine, and its antagonistic effects on important rice pathogens were assessed and its antimicrobial active substances were preliminarily determined and analyzed.

## Materials and methods

### Bacterial strains isolation from Chinese herbal medicine

The antagonistic microbial strains were deliberately isolated from the traditional Chinese medicines, i.e., *Bolbostemmatis Rhizoma*, *Radix isatidis*, and *Flos Lonicerae*. *Bolbostemmatis Rhizoma* (Tubeimu) is the dried tuber of *Bolbostemma paniculatum* (Maxim.) Franquet, belonging to Cucurbitaceae family (Liu et al., 2004). *Radix isatidis* (Banlangen) is the dried root of *Isatis indigotica*, a biennial herbal plant belonging to Cruciferae. *Flos Lonicerae* (Jinyinhua) is the flower bud and early flower of *Lonicera japonica* Thund., which is a semi-evergreen and perennial woody climbing shrub belonging to the Caprifoliaceae family. In this study, the samples *Bolbostemmatis Rhizoma*, *Radix isatidis*, and *Flos Lonicerae* were collected from Huyi District, Xian City, Shanxi Province, China (33°46′N, 108°22′E), Anguo City, Hebei Province, China (38°42′N, 115°33′E), and Jingxi County, Guangxi Province, China (23°13′N, 106°42′E), respectively.

The collected Chinese herbal materials were cut into small segments of 1 cm in size, sterilized on the surface, smashed and ground in a sterile mortar containing 10 mL sterile water, and then put into a triangular flask containing 90 ml sterile water. The bacterial suspension was prepared by shaking culture at 200 rpm for 30 min. 1 mL bacterial suspension was diluted with sterile water to concentrations of 10^–2^, 10^–3^, and 10^–4^, respectively. Dip 100 μL diluted bacterial solution evenly spread on agar plates of NA medium (Supplementary Table 1), repeat 3 times. After 48 h culture at 28°C, single colonies with different shape were selected for subculture.

### Rice and pathogens

Rice (*Oryza sativa* L.) cultivar Nipponbare was used as a host for the respective pathogens including *Xoc*, *Xoo*, *M. oryzae*, *R. solani*, *F. fujikuroi*, and *U. virens* in this study (Table 1).

**TABLE 1:** Bacterial and fungal strains used in this study.

Strain	Relevant character	Source
*Bacillus velezensis* BR-01	Wild-type, isolated from *Bolbostemmatis Rhizoma* in Huyi district from Shanxi province of China	This study
*Magnaporthe oryzae* GM05	Causing rice blast	This lab*
*Ustilaginoidea virens* GU03	Causing rice false smut	This lab
*Fusarium fujikuroi* GF02	Causing rice bakanae disease	This lab
*Rhizoctonia solani* GR06	Causing rice sheath blight	This lab
*Xanthomonas oryzae* pv. *oryzicol* GX01	Causing rice bacterial leaf streak	This lab
*Xanthomonas oryzae* pv. *oryzae* PXO99^A^	Causing rice bacterial blight	This lab

*State Key Laboratory for Conservation and Utilization of Subtropical Agro-Bioresource.

*Xoc* and *Xoo* strains were grown in NA medium at 28°C. *U. virens* was grown on potato sucrose agar medium (PSA) (Supplementary Table 1) at 28°C. *M. oryzae*, *R. solani*, *F. fujikuroi*, and *U. virens* were cultivated on potato dextrose agar medium (PDA) (Supplementary Table 1) at 28°C.

### Initial screening of antagonistic bacteria

Given that there have been considerable reports about *B*. *velezensi*s biological control of *M*. *oryzae*, the rice fungal pathogen, in this study, *X*. *oryzae* pv. *oryzicola*, the rice bacterial pathogen, were deliberately chosen as the initial control target indicator.

The isolated antagonist bacteria and *Xoc* were inoculated in NB medium (Supplementary Table 1), and cultured in a shaker at 28°C, respectively. After overnight culture at 200 rpm, the bacterial concentration was adjusted to OD_600_ = 1.0.

Firstly, a layer of 5 ml water agar was placed at the bottom of the petri dish to ensure that the bottom of the petri dish was smooth. After cooling, an Oxford cup was placed in the middle of the petri dish. The completely melted NA agar medium was cooled to about 45°C, and 1 mL indicator bacteria liquid was added to each 100 mL medium. After shaking evenly, it was poured into the petri dish, about 15 ml per dish. After cooling, the Oxford cup was taken out, and 0.1 mL antagonistic bacteria liquid was added to each hole. The same amount of NB medium was added as the blank control, with three repeated treatments. Inverted culture at 28°C for 2 days, the diameter of inhibition zone was observed and measured (Li et al., 2019).

### Evaluation of antagonistic spectrum of the antagonistic bacterium

For determination of the antagonistic spectrum of the antagonistic bacterium, the pathogens of the major rice diseases, i.e., *Xoo*, *M. oryzae*, *R. solani*, *F. fujikuroi*, and *U. virens*, were used as targeted indicators (Table 1), after the initial screening.

Almost same procedures in the antagonistic test on *Xoc* were used on *Xoo*. Determination of antagonistic spectrum against pathogenic fungi were carried out by using plate confrontation method (Pastrana et al., 2016). The indicator fungi block was inoculated in the center of PDA plate with a puncher of 5 mm in diameter, and 1 μL of antagonistic bacterial suspension (OD_600_ = 1.0) was dotted around the indicator fungi. The dotted point was 2 cm away from the fungi block. The plates inoculated with the same amount of NB medium were taken as the control group, and the treatment was repeated for three times. Inverted culture in a constant temperature incubator at 28°C, when the diameter of the pathogen in the control group grew to about 3/4 of the diameter of the petri dish, the diameter of the pathogen was measured by Cross-measurement method (Yang et al., 2017), and the antifungal rate of the strain was calculated according to the following formula.


(1) $$I=\frac{d_{0}-d_{1}}{d_{0}-d}\,\text{×}\,100\%$$

Where *I* = antifungal rate; *d*_0_ = diameter of the control group; *d*_1_ = diameter of the treatment group; *d* = diameter of the punch.

### Physiological, biochemical, and molecular characterization

The antagonistic bacteria were primarily identified by observing the morphology, size, color, wet or dry, smooth or rough of individual colonies according to the methods of Berger’s Manual of Bacterial Identification (Holt et al., 1994) and Common Bacterial Systems Identification Manual (Dong and Cai, 2001). A number of physiological and biochemical characterization tests such as Gram staining, contact enzyme, nitrate reduction, liquefaction of gelatin, hydrogen sulfide production, carbon source utilization, V-P (Voges–Proskauer) test and MR (methyl red) test were performed on antagonistic bacteria. The treatment was repeated three times.

Genomic DNA was extracted and purified using the whole genome DNA sequencing kit (Tiangen Biochemical Technology Co., Ltd., Beijing, China). 16S rDNA and *gyrB* genes of the antagonistic bacterium were amplified by primer pairs 27F (5′-AGAGTTTGATCCTGGCTCAG-3′), 1492R (5′-GGTTACCTTGTTACGACTT-3′) (Mahmood et al., 2006) and UP1 (5′-GAAGTCATCATGACCGTT CTGCAYGCNGGNGGNAARTTYGA-3′), UP2r (5′-AGCAG GATACGGATGTGCGAGCCRTCNACRTCNGCRTCNGTCAT-3′) (Yamamoto and Harayama, 1995), respectively.

The PCR amplification system was 50 μL of 5 × PCR buffer 10 μL, primer F 1.2 μL, primer R 1.2 μL, 2.5 mM dNTPs 5 μL, DNA template 1 μL, DNA polymerase 0.6 μL, and add ddH_2_O to 50 μL. PCR cycle conditions: denaturation at 95°C for 30 s, annealing at 60°C for 30 s, extension at 72°C for 30 s, a total of 30 cycles; then the products were kept at 72°C for 10 min and detected by 1% agarose gel electrophoresis. The amplified products were sequenced by Nanning Qingke Biotechnology Co., Ltd. The sequencing results were compared with the known sequences in NCBI database by nucleotide BLAST, and the phylogenetic tree was constructed by N-J method in MEGA 11.0.

### Detection of antagonist related lytic enzymes and secondary metabolites

The activities of protease, cellulase, glucanase, and chitinase of BR-01 were detected on agar plates containing skim milk, sodium carboxymethyl cellulose, β-glucan, and colloidal chitin, respectively (Ghose, 1987; Roberts and Selitrennikoff, 1988; Shakeel et al., 2015; Li et al., 2018). The antagonistic bacterial suspension (1 μL, OD_600_ = 1) was inoculated on four testing media in plates (Supplementary Table 1) and cultured at 28°C for 2–4 days. The ability of antagonistic bacterium to produce various hydrolytic enzymes was judged by observing the growth of bacterial colonies and the size of hydrolytic circles around colonies. The hydrolysis circle of cellulase was first stained with Congo red and then observed.

Chrome-azurol S (CAS) medium (Supplementary Table 1) was used to detect the ability of antagonistic bacterium to produce siderophore. The antagonistic bacterial suspension (1 μL, OD_600_ = 1) was inoculated on CAS medium plates and cultured at 28°C for 2–4 days. The yellow-orange halo around the colonies was considered to be caused by the presence of siderophore (Schwyn and Neilands, 1987).

The ability of antagonistic bacterium to produce IAA was determined by observing whether the fermentation supernatant of YMB medium (Supplementary Table 1) mixed Salkowski reagent will appear obvious red (Gordon and Weber, 1951).

To check whether antagonistic bacterium can be continuously passaged three times in ADF medium (Supplementary Table 1) to determine whether antagonistic bacterium can produce ACC deaminase (Penrose and Glick, 2003).

### Detection of synthesis-related genes for antimicrobial peptides

The antimicrobial lipopeptide synthesis-related genes were detected by using specific primers (Mora et al., 2011; Plaza et al., 2015; Yang et al., 2015; Sajitha and Dev, 2016) listed in Supplementary Table 2. The PCRs were carried out using approximately 0.5 μg of total bacterial DNA (1 μL), 10 μL of 5× PCR buffer, 5 μL of dNTPs (2 mM), 1.5 μL of each primer (10 μM), 0.6 μL of DNA polymerase, and adequate ddH_2_O so that the final volume of the mixture was 50 μL. The PCR mixtures were denatured at 95°C for 5 min, which was followed by 30 cycles of 94°C for 30 s, 51°C∼60°C for 30 s, and 72°C for 1 min and then a final extension at 72°C for 5 min. PCR products were checked for sizes by electrophoresis on a 1.5% agarose gel.

### Preparation of culture extract and its antimicrobial activity test

In this study, the antimicrobial substance from cell-free supernatants of *B. velezensis* BR-01 was purified using phosphate-buffered saline (PBS) (Supplementary Table 1) and methanol extraction after ammonium sulfate precipitation at 60% saturation (Xu et al., 2018). The methanol extract was dried under reduced pressure in a rotating evaporator at 43°C to obtain the dry powder of antibacterial peptide crude extract (Jasim et al., 2014).

The inhibitory sample was prepared by dissolving 3 mg crude extract in 1 ml methanol and filtered through 0.22 μm filter (Yang Y. Y. et al., 2021). *Xoc* and *Xoo* were used as indicator bacteria and the antibacterial activity was detected by Oxford cup method. *M*. *oryzae* and *U*. *virens* were used as indicator fungi, and the inhibitory samples were mixed with PDA medium at a ratio of 1:40 by radial growth assay (Pastrana et al., 2016). With the addition of the same amount of methanol as the control, each treatment was repeated three times. For antagonistic to bacterial pathogens, the treatment and control groups were cultured at 28°C for 2 days. For antagonistic to fungal pathogens, the treatment and control groups were cultured at 28°C, when the diameter of the fungal colonies in the control group grew to about 3/4 of the diameter of the petri dish, the diameter of the pathogen was measured by Cross-measurement method (refer to 1.4) (Yang et al., 2017).

### Compound identification from culture extract using liquid chromatograph-mass spectrometer

Liquid Chromatograph-Mass Spectrometer (LC-MS) analysis was performed to identify purified bioactive substances in crude extract from *B. velezensis* BR-01 (Xu et al., 2018). Using Thermo Xcalibur 4.0 software, the relative molecular mass of the target substance was measured according to the mass-to-charge ratio (m/z) of the sample, and the types of lipopeptides produced by the strain were preliminarily determined by comparison (Xia et al., 2019).

Chromatographic conditions: ACQUITY UPLCBEHC18 column (50 mm × 2.1 mm, 1.7 μm); in the positive ion (ESI+) mode, the mobile phase A was 0.1% formic acid water, and B was methanol. Sample gradient elution program: 0∼2 min, 95% A; 2∼13 min, 95% A∼5% A; 13∼16 min, 5% A; 16∼16.1 min, 5% A∼95% A; 16.1∼18 min, 95% A. The flow rate was 0.3 mL/min, and the injection volume was 2 μL.

Mass spectrum conditions: The ion source is spray electrothermal spray (HESI) with a temperature of 300°C. The spray voltage is 3.0 kV under positive ion mode. The temperature of transmission capillary is 320°C, the sheath gas is 30 psi, and the auxiliary gas flow velocity is 10 psi; the scanning mode is full MS/dd-MS2, the quality range is 200∼2,000 *m/z*, and the resolutions of primary scanning and secondary scanning are 70,000 and 17,500, respectively. Collision gas: high purity nitrogen.

### Biocontrol efficacy assays under greenhouse conditions

Biocontrol efficacy assays were carried out in a greenhouse at constant temperature of 28°C and relative humidity of 90%. 45-day-old rice plants in pots were used in infection challenges. *Xoc*, *Xoo*, and *B. velezensis* BR-01 strains were inoculated into NB medium at 28°C and cultured at 200 rpm for 18 h until logarithmic growth period, collected and then diluted with NB medium to OD_600_ = 0.5. The infiltration method was used for inoculation of *Xoc*, by which the *Xoc* culture suspension was infiltrated into rice leaves using needleless syringe (Yang and Bogdanove, 2013). The leaf-cutting method was used for inoculation of *Xoo*, by which rice leaves were clipped using scissors dipped in *Xoo* culture suspension (Ke et al., 2017).

Four different treatment groups were set up (Supplementary Table 4; Li et al., 2019; Huang et al., 2021). CK group: inoculated with rice pathogenic bacteria, 12 h after inoculation spray NB medium as control treatment. NB medium group: replace the inoculated bacterial suspension with NB medium and spray NB medium 12 h after inoculation. Treatment group: inoculated with rice pathogenic bacteria, 12 h after inoculation spray *B. velezensis* BR-01 bacterial suspension. Preventive group: inoculated with rice pathogenic bacteria, 12 h before inoculation spray *B. velezensis* BR-01 bacterial suspension. Each treatment group of each rice pathogenic bacterium was inoculated with 10 rice plants and 5 leaves per plant. About 10 days after inoculation, the expansion of the lesions was observed and recorded, and the control effect was calculated according to the formula (Li et al., 2019).


(2) $$C=\frac{L_{1}-L_{2}}{{\text{L}}_{1}}\,\text{×}\,100\%$$

Where C = control effect; L_1_ = length of spots in control group; L_2_ = length of spots in treatment group.

### Data analysis

Each experiment was independently repeated at least three times. The software SPSS v20.0 (SPSS, Chicago, IL, USA) was used for statistical analysis. Statistical significance between multiple groups was determined using analysis of variance (ANOVA). All the results were expressed as mean ± SD (*n* = 3). *P* < 0.05 were considered statistically significant.

## Results and analysis

### Isolation of antagonistic bacteria and evaluation of their inhibition effects

**FIGURE 1:**
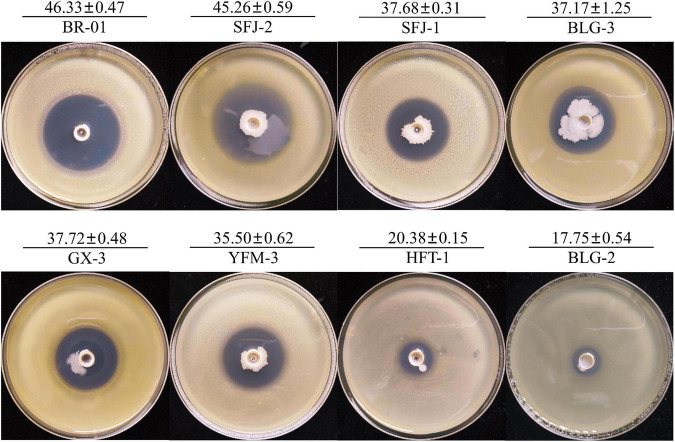
Antagonistic effect of antagonistic bacteria on *Xanthomonas oryzae* pv. o*ryzicola.* Values given are the means ± standard deviations of triplicate measurements. The unit of measurement for inhibition zones is millimeter.

**FIGURE 2:**
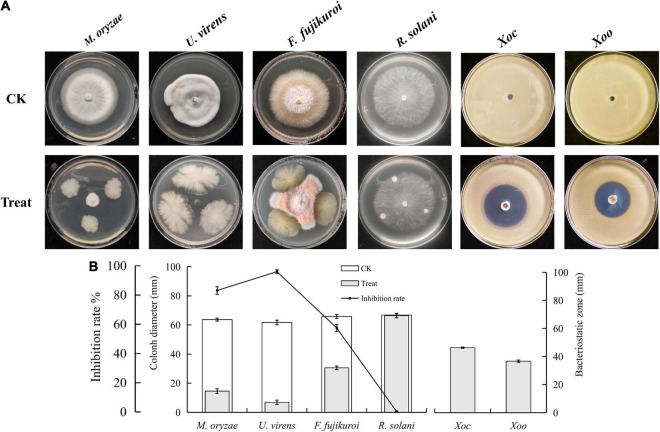
**(A)** Antagonistic effects of BR-01 against a variety of rice pathogens and **(B)** data statistics. Inhibition rate of BR-01 on pathogenic fungi was measured when pathogenic fungi in control group grew to about 3/4 of culture dish. Values given are the means ± standard deviations of triplicate measurements.

To screen the microbial resources for controlling various diseases of rice, three kinds of Chinese herbal medicines were collected from three provinces in China. Eight bacterial strains were isolated, including three strains from *Bolbostemmatis Rhizoma*, three strains from *Radix isatidis*, and two strains from *Flos Lonicerae*, which had antagonistic effects on *Xoc* in the Oxford cup assays (Figure 1). Among them, the strongest antagonistic strain was isolated from *Bolbostemmatis Rhizoma*, with an average inhibition zone diameter of 46.33 mm, designated BR-01. The plate co-culture test showed that BR-01 had antagonistic activity against *M. oryzae*, *U. virens*, and *F. fujikuroi*, and the relative inhibition rates of mycelial growth were 83.71, 96.65, and 57.92%, respectively, but had no significant antagonistic effect against *R. solani*. The results of the Oxford cup method showed that BR-01 also had a strong antagonistic effect against Xoo, with an average inhibition zone diameter of 36.70 mm (Figure 2).

### Characterization of antagonistic bacterium BR-01

**FIGURE 3:**
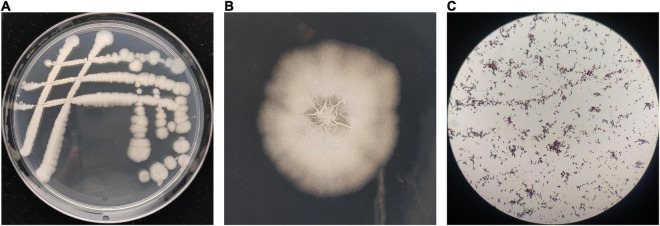
Colony morphological characteristics of BR-01. **(A)** Appearance of antagonistic bacterium BR-01 on NA medium. **(B)** Antagonistic bacterium BR-01 single colony morphology. **(C)** Gram staining of antagonistic bacterium BR-01.

The morphological observation showed that the single colonies of BR-01 were white, flat, opaque, rough, and wrinkled with irregular edges on the NA medium, and Gram staining was positive (Figure 3). Physiological and biochemical characterization showed that BR-01 could utilize sucrose, D-glucose, D-maltose, and D-mannitol. Contact enzyme reaction, gelatin hydrolysis, and starch hydrolysis were positive reactions. Hydrogen sulfide production, citrate utilization, and phenylalanine dehydrogenase test were negative reactions (Table 2). According to the physiological and biochemical characteristics of BR-01, combined with the morphological characteristics of colonies, and referring to Bergey’s universal methods (Holt et al., 1994), BR-01 was preliminarily identified as *Bacillus* sp.

**TABLE 2:** Physiological and biochemical characteristics of strain BR-01.

Physiological and biochemical project	Result	Physiological and biochemical project	Result
Contact enzyme reaction	+	V-P reaction	−
Sucrose utilization	+	MR reaction	−
D-glucose utilization	+	Hydrolyzed gelatin	+
D-maltose utilization	+	Hydrolyzed starch	+
D-mannitol utilization	+	Hydrogen sulfide production	−
D-sorbitol utilization	+	Citrate utilization	−
α-lactose utilization	+	Nitrate reduction reaction	+
D-galactose utilization	+	Amphetamine dehydrogenase	−
D-fructose utilization	+	Hydrolyzed casein	+

“+”: Positive reaction; “−”: Negative reaction.

**FIGURE 4:**
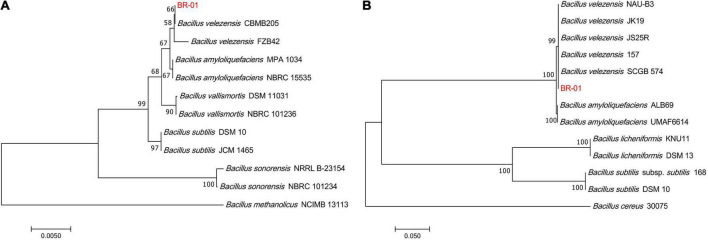
Neighbor-joining phylogenetic tree of the antagonistic bacterium BR-01 were constructed by MEGA 11.0. **(A)** Based on 16S rDNA gene. **(B)** Based on *gyrB* gene. The numbers at the branches indicate the confidence level calculated by bootstrap analysis (1000). The scale bar shows the evolutionary distance between species.

To clarify the taxonomic status of BR-01, its 16S rDNA sequence was analyzed by BLAST in NCBI. It was found that it had the highest homology with *B. velezensis* CBMB205 (NR116240.1) and the identity was 99.58%. BLAST analysis of the *gyrB* gene sequence of BR-01 also showed that it had 100% identity with the *gyrB* gene of *B. velezensis* JS25R (NZ CP009679.1). Using MEGA 11.0 software, phylogenetic trees based on 16S rDNA and *gyrB* gene sequences were constructed, respectively. The results showed that the 16S rDNA and *gyrB* sequences of BR-01 were clustered into one branch with *B*. *velezensis* (Figure 4). Thus, the antagonistic strain BR-01 was identified as *B. velezensis* by combining the morphological characteristics, physiological and biochemical characteristics, and sequence analysis results.

### Detection of antagonist related lytic enzymes and secondary metabolites from BR-01

**FIGURE 5:**
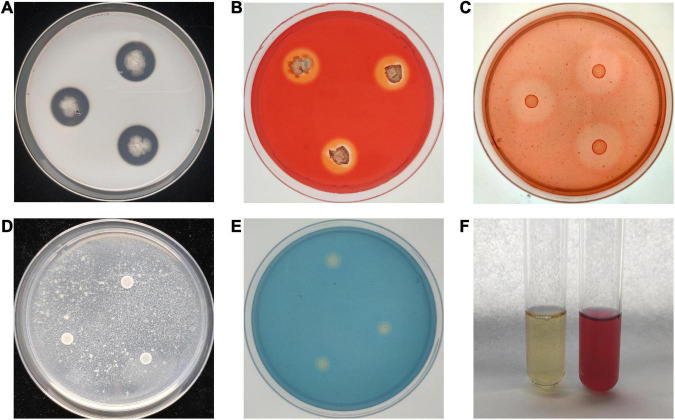
Detection of antagonistic enzymes and secondary metabolites in *B. velezensis* BR-01. **(A)** Protease detection; **(B)** cellulase detection; **(C)** β-1,3-glucanase detection; **(D)** chitinase detection; **(E)** siderophore detection; **(F)** indole-3-acetic acid (IAA) detection.

To study the antagonistic mechanism of *B. velezensis* BR-01, We examined the ability of *B. velezensis* BR-01 to produce antagonist related lytic enzymes and secondary metabolites. Translucent hydrolysis circles were formed around all colonies of *B. velezensis* BR-01 on the medium for protease, cellulase and β-1,3-glucanase assays, indicating that *B. velezensis* BR-01 could produce protease, cellulase and β-1,3-glucanase. In addition, *B. velezensis* BR-01 grew well on the medium with chitin as the sole carbon source, so it was considered to have the ability to produce chitinase, although there was no obvious hydrolysis circle around the colony of *B. velezensis* BR-01. The study on the production of fungal cell wall degrading enzymes by *B. velezensis* BR-01 can explain the swelling of fungal cells and the loss of cell wall integrity to a certain extent, leading to cell lysis and death (Muleta et al., 2007), providing a basis for the further development and application of *B. velezensis* BR-01 (Figure 5 and Table 3).

**TABLE 3:** Detection of antagonistic related products of BR-01.

Enzymes and secondary metabolites	Result
Protease	+
Cellulase	+
β-1,3-glucanase	+
Chitinase	+
Siderophore	+
Indoleacetic acid	+
ACC deaminase	+

+, the production of the biocontrol-related substance by the test strain.

Compared with the control group, the color of the YMB liquid medium fermentation supernatant of *B. velezensis* BR-01 was significantly red after mixing with Salkowski reagent, indicating that *B. velezensis* BR-01 could synthesize IAA using tryptophan as the precursor. *B. velezensis* BR-01 could be continuously passaged three times on ADF medium with ACC as the sole nitrogen source, indicating that *B. velezensis* BR-01 could produce ACC deaminase. *B. velezensis* BR-01 could change CAS medium from blue to light orange, indicating that it could produce Siderophore. IAA is a plant auxin that can effectively promote plant growth and affect plant physiological metabolism. ACC deaminase can degrade ethylene precursor ACC, thereby reducing ethylene levels during plant growth and contributing to plant growth (Nascimento et al., 2014). Siderophore absorb ferric ion in the surrounding environment and provide more ferric ion to plants for their use, so that plant pathogenic microorganisms lack iron nutrition and cannot grow and reproduce (Wang et al., 2014). *B. velezensis* BR-01 could promote plant growth by releasing these substances and indirectly improve plant disease resistance.

### Identification of the synthetic genes for antimicrobial peptides in BR-01

**FIGURE 6:**
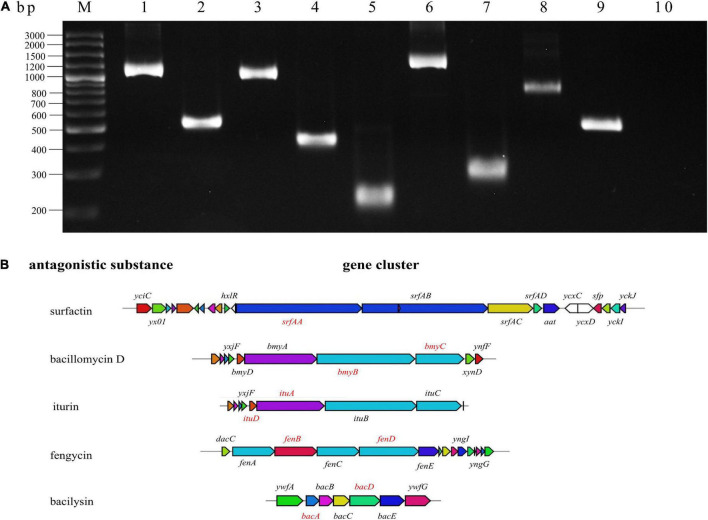
Amplification of antimicrobial peptide synthesis gene of *B. velezensis* BR-01. **(A)** Detection of antimicrobial peptide biosynthetic gene in *B. velezensis* BR-01 by PCR. M, DNA marker; 1, *ituA*; 2, *ituD*; 3, *bmyC*; 4, *bmyB*; 5, *srfAA*; 6, *fenB*; 7, *fenD*; 8, *bacD*; 9, *bacA*; 10, non-template control. **(B)** Five antimicrobial peptide biosynthetic gene clusters. The red gene denote the successful antibacterial peptide biosynthesis gene amplified by PCR. Pictures of antimicrobial peptide gene cluster were from antiSMASH (http://antismash.secondarymetabolites.org/).

*Bacillus velezensis* can produce a variety of antimicrobial peptides. To identify the synthetic genes for antimicrobial peptides in BR-01, the target genes involving in the synthesis of iturin, bacillomycin, surfactin, fengycin, and bacilysin were subjected to be amplified with specific primers for certain genes using the genomic DNA of *B. velezensis* BR-01 as the template (Chen et al., 2009; Sumi et al., 2015; Yang and Du, 2022). The PCR results showed that BR-01 might contain *ituA and ituD* genes related to iturn synthesis, *bmyB* and *bmyC* genes for bacillomycinD synthesis, *srfAA* for surfactin synthesis, *fenB* and *fenD* fengycin synthesis, and *bacA* and *bacD* involving in bacilysin synthesis (Figure 6), indicating that *B. velezensis* BR-01 has the potential to produce the common antimicrobial peptides in *B. velezensis*.

### Antagonistic activity test and liquid chromatograph-mass spectrometer preliminary identification of BR-01 culture extract

**FIGURE 7:**
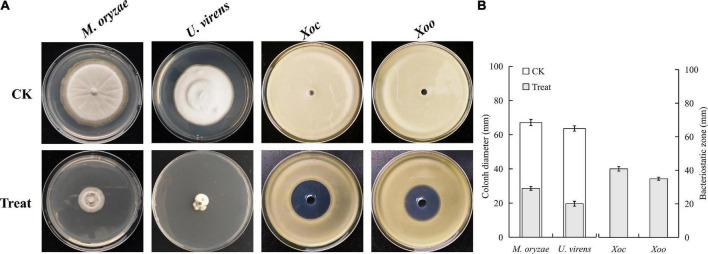
**(A)** Antibacterial activity test of *B. velezensis* BR-01 culture extract and **(B)** data statistics. Fungal plate photographs were taken when pathogenic fungi in the control group grew to about 3/4 of the culture dish. Bacterial plate photographs were taken after 2 days of culture. All plates were placed at 28°C.

In this study, the antimicrobial peptide crude extract was successfully extracted from cell-free supernatants of *B. velezensis* BR-01. The results of the antagonistic activity assay of the antibacterial peptide crude extract showed that when an appropriate amount of crude extract methanol solution was added to the medium, the growth of the four indicator microbes was inhibited to varying degrees, indicating that crude extract of antimicrobial peptide from *B*. *velezensis* BR-01 has an antagonistic effect (Figure 7).

The crude extract of antimicrobial peptide from *B. velezensis* BR-01 was analyzed by LC-MS detection and analysis technology (Xu et al., 2018). Studies have shown that antimicrobial peptides metabolized by *Bacillus* mainly include iturin, fengycin, and surfactin. Their specific composition and molecular weight are known. Combined with the peaks appearing in each time period of the chromatogram corresponding to the mass spectrum, a series of single peaks in the mass spectrum were analyzed according to the relative molecular mass and the structural characteristics of the “-CH2-” fat chain (Ma and Hu, 2019; Xia et al., 2019; Hasan et al., 2020). The mass spectra in the range of m/z 1000–2000 were mainly as follows: (1) m/z 1008, m/z 1022, m/z 1036, and m/z 1050 were speculated to be surfactin homologues; (2) m/z 1044, m/z 1058, m/z 1072, m/z 1086, and m/z 1100 were speculated as iturin homologues; (3) m/z 1463, m/z 1477, and m/z 1491 were speculated to be fengycin homologues. Based on the above results, it was speculated that the strain might produce three types of lipopeptide antibiotics: surfactin, iturin, and fengycin (Supplementary Figure 1 and Supplementary Table 3).

### Biocontrol efficacy of BR-01 on rice bacterial streak and bacterial leaf blight

**FIGURE 8:**
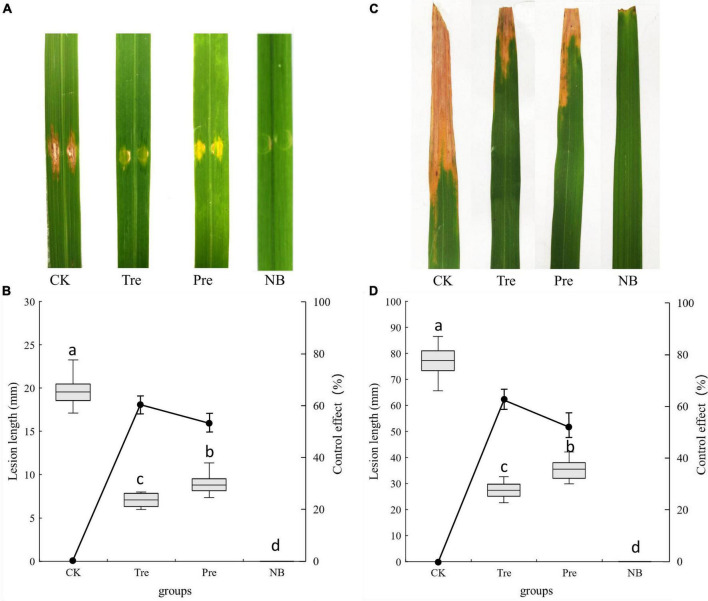
Effect of *B. velezensis* BR-01 on inhibition of rice bacterial leaf streak and rice bacterial blight under greenhouse conditions. **(A)** Representative lesions on rice leaves of rice bacterial leaf streak. **(B)** Lesions length of rice bacterial leaf streak and biocontrol effect of *B. velezensis* BR-01. **(C)** Representative lesions on rice leaves of rice bacterial blight. **(D)** Lesions length of rice bacterial blight and biocontrol effect of *B. velezensis* BR-01. Different lowercase letters represented significant difference (*P* < 0.05).

Under greenhouse conditions, the biological control effects of *B. velezensis* BR-01 on rice bacterial leaf streak and rice bacterial blight were determined (Li et al., 2019; Huang et al., 2021). After 10 days of inoculation, obvious lesions were found on the leaves of rice inoculated with *Xoc* and *Xoo*, respectively. The control effects of spraying *B. velezensis* BR-01 fermentation broth on rice bacterial leaf streak and rice bacterial blight in greenhouse were determined. The results showed that after spraying *B. velezensis* BR-01 fermentation broth, the incidence of the treatment group and the prevention group was significantly lower than that of the control group, and the control effects of the treatment group on rice bacterial leaf streak and rice bacterial blight were 64.19 and 66.16%, respectively. The control effects of *B. velezensis* BR-01 fermentation broth on rice bacterial leaf streak and rice bacterial blight in the prevention group were 56.54 and 55.41%, respectively, indicating that *B. velezensis* BR-01 fermentation broth had obvious control effects on rice bacterial leaf streak and rice bacterial blight (Figure 8).

## Discussion

With the improvement of low-carbon and sustainable agriculture, biological approaches are urgently needed to minimize crop yield losses resulting from pest activity and reduce impacts of pest management on human health and the environment (Baker et al., 2020). Rice, one of the most important staple crops, is especially concerned owing to its huge chemical consumption, including pesticides, plant growth regulators and chemical fertilizers. The biological control of rice diseases is particularly important both on economic and ecological aspects in the present world fraught with potential food crises.

Considering the vulnerability of rice to the infection of a variety of diseases, in this study, a *B. velezensis* BR-01 was isolated from *Bolbostemmatis Rhizoma*, a traditional Chinese medicine. The confrontation culture and Oxford cup assays demonstrated that *B. velezensis* BR-01 had strong inhibitory effect on *M. oryzae*, *U. virens*, *F. fujikuroi*, *Xoc*, and *Xoo*, five major rice pathogens but *R. solani* (Supplementary Table 4). *B. velezensis* BR-01 could produce protease, cellulase, β-1,3-glucanase, chitinase, indoleacetic acid, siderophore, and ACC deaminase, and might produce three lipopeptide antibiotics, surfactin, iturin, and fengycin based on LC-MS results. Furthermore, the plant assays showed that *B. velezensis* BR-01 had significant control effects on rice bacterial leaf streak and rice bacterial blight by pot experiments in greenhouse. To our knowledge, this is the only report about a *B. velezensis* strain which has a broad-spectrum against five major rice diseases. It was reasonably to expect reducing the frequency of farming operations using the multiple antagonistic agents.

Although the antagonistic efficacies between the different biocontrol agents were not compliant to be quantitively compared when the individual tests were not conducted on the same conditions, from the comparative literature analysis, we might still evaluate *B. velezensis* BR-01 to have the potential being a strong and broad-spectrum biocontrol agent in rice disease control. Currently, there have been numerous reports about the antagonistic effects and biocontrol efficacies of *B. velezensis* strains *on M*. *oryzae*, some of which were registered as the bacterial pesticides used in rice production (Chen et al., 2021). From our dual culture tests, *B. velezensis* BR-01 has an 83.7% relative inhibition rate on *M*. *oryzae*, which is comparable to most of other *B. velezensis* strains on *M*. *oryzae* (Sha et al., 2020; Chakraborty et al., 2021). *B. velezensis* BR-01 also showed antifungal activity against *U. virens* and *F. fujikuroi*, with relative inhibition rates 96.6 and 57.9%, respectively. *U. virens* is the causal agent of rice false smut disease, one of the most devastating rice fungal diseases in China (Miao et al., 2020). Rice false smut disease not only causes severe yield loss and grain quality reduction, but also threatens food safety due to its production of mycotoxins. *U. virens* specifically infects rice panicles before heading. The hyphae colonize in florets, and eventually form rice false smuts with powdery chlamydospores on rice grains (Fan et al., 2016; Baite et al., 2022). Once the disease occurs, it is difficult to control. *F. fujikuroi* is a prevalent plant pathogen, which causes the bakanae disease of the rice plant (Hossain et al., 2016). The infection sites of bakanae disease and false smut are totally different, however, their latent periods in the disease courses are long and intangible, which might result in inconvenience to precise prevention and control by using chemicals or fungicides. It is highly expected that biological agents might play important roles in preventing bakanae disease and false smut in niche competitions (Hossain et al., 2016; Baite et al., 2022).

As to rice bacterial diseases, *B. velezensis* BR-01 was demonstrated to have strong antagonistic effects on *Xoc* and *Xoo* on plate confrontation tests, and have the significant biocontrol efficacies on rice bacterial leaf streak and leaf blight in the pot experiments. Previously, some other *B. velezensis* strains have been reported having inhibitory effects on *X. oryzae*, for example, strain FZB42 isolated from the rhizosphere soil of lettuce (Wu et al., 2015), strain 504 from water spinach rhizosphere soil (Li et al., 2019), and HN-2 from soil (Jin et al., 2020), which were emphasized specifically antagonistic on *Xoo* and *Xoc* of rice pathogens. Recently, strain SF327 isolated from orchard soil has been demonstrated with functions of promoting plant growth and the inhibitory effects on three rice pathogens, i.e., *M. oryzea*, *Xoo*, and *Xoc* (Fang et al., 2022). The relative inhibition rate of *B. velezensis* BR-01 on *Xoo* PXO99^A^ is similar to that of SF27 on *Xoo* PXO99^A^, which provided a strong support for biocontrol effect of *B. velezensis* BR-01 on rice bacterial diseases.

To data, there are several isolation approaches for *B. velezensis* strains used in rice disease control. First, soil microorganisms (including paddy field and non-paddy field). Soil is the “natural medium” of microorganisms and the most abundant strain resource pool (Wu et al., 2015; Jin et al., 2020; Fang et al., 2022). The second is plant rhizosphere bacteria. Because plant rhizosphere growth promoting bacteria (PGPR) have strong rhizosphere colonization ability and unique microbiological functions, they have become a hot spot in the research of biocontrol microorganisms (Liu et al., 2018). Plant endophytes are also an important source of biocontrol microorganisms. Their stable living space and strong colonization ability make them have excellent characteristics as biocontrol factors of plant diseases (Wen et al., 2004; Hossain et al., 2016). In addition, microorganisms in complex habitats (such as marine, lake, and river) show great potential as biocontrol factors of plant diseases and producers of bioactive substances because of their unique biodiversity. The *B. velezensis* isolates from different environments showed different antagonistic spectrum and growth promoting functions. It is reasonable to attribute the diversities of biological functions of certain bacteria to their different environmental adaptation, which prompts to isolate and screen more versatile antagonistic bacteria from different habitats.

In this study, strains were isolated from Chinese medicinal plants different from most crops for screening biocontrol microorganisms. Medicinal plants are a group of plants with special chemical composition and physiological functions, and are one of the main raw materials for screening natural medicinal ingredients (Lv et al., 2017). Because endophytes form a friendly kinship with host plants in the long-term coevolution process, some endophytes have the ability to produce the same or similar bioactive compounds as their host plants (Zhao et al., 2011; Lv et al., 2017). Therefore, in general, endophytic bacteria contained in plants with medicinal activity are more likely to produce antibiotic activity (Deng and Yang, 2010). Ding et al. (2010) analyzed the secondary metabolites of endophytic bacteria from the tissue of a *Bruguiera Gymnorrhiza*. For the first time, the anti-HIV activity of the indole semiterpenoid Xiamycin was obtained from bacteria. Chen et al. (2013) isolated a strain of *B. amyloliquefaciens* from *Ophiopogon japonicus*. The exopolysaccharide secreted by it has antitumor activity. Wang et al. (2021) isolated a *pseudomonas fluorescens* strain from the *Bletilla striata*, which had inhibitory effects on *Staphylococcus aureus*, *Escherichia coli*, *Micrococcus luteus*, and *Pectobacterium carotovorum* subsp. *carotovorum*. It was speculated that strain BSR2010 inhibited the growth of four pathogens by producing 2,4-diacetylphloroglucinol (2,4-DAPG). These studies indicate that endophytic bacteria of medicinal plants can produce a wide variety of secondary metabolites with novel structures, from which new antibacterial, antiviral, and antitumor active substances can be screened. The exploitation and utilization of endophytic bacteria in medicinal plants has potential theoretical and practical value.

*Bolbostemmatis Rhizoma* is the dry tuber of *Bolbostemma paniculatum* (Maxim.) Franquet, which contains triterpenoids, sterols and glycosides, anthraquinones, and alkaloids. Triterpenoids from *Bolbostemmatis Rhizoma* have anti-tumor, anti-inflammatory, and antiviral pharmacological effects (Yang Z. H. et al., 2021). So far, no reports have been reported on the isolation of endophytic bacteria from *Bolbostemmatis Rhizoma* with antagonistic activity against rice pathogens. In this study, endophytic bacteria were isolated from the tubers of *Bolbostemma paniculatum* (Maxim.) Franquet, and their antibacterial activities against a variety of rice pathogens were studied, to screen out excellent strains with broad-spectrum and high antibacterial activity, and then study the antibacterial components of their metabolites, so as to lay the foundation for the further development of *Bolbostemmatis Rhizoma* endophytic bacteria resources.

In conclusion, all parameters tested *in vitro* of strains isolated from *Bolbostemmatis Rhizoma* of *B. velezensis* RB-01 showed that they could antagonize a variety of major rice pathogens. This indicates that *B. velezensis* RB-01 is a new microbial resource for biological control of various rice diseases.

## Data availability statement

The original contributions presented in this study are included in the article/Supplementary material, further inquiries can be directed to the corresponding authors.

## Author contributions

Y-QH, JZ, and WJ conceived and designed the research and drafted the manuscript. JZ, YX, YL, and SL isolated and identified the bacterial strains. JZ, XL, and YML isolated the genome DNA and performed the PCR analysis. JZ, XM, and YX conducted the plant assays. JZ, SML, and YL carried out the LC-MS assays. YL, FL, and WJ conducted bioinformatical and statistical analysis. WJ and YX contributed to revising the manuscript. All authors contributed to the interpretation of data and approved the final manuscript.
