# Supplementary material for: Characterization of a Bacillus velezensis strain isolated from Bolbostemmatis Rhizoma displaying strong antagonistic activities against a variety of rice pathogens
Source: Front Microbiol. 2022 Sep 28;13:983781. doi: 10.3389/fmicb.2022.983781 (PMC9555170; doi:10.3389/fmicb.2022.983781)
Supplement: Supplementary file 1 [file Data_Sheet_1.docx]

**Supplementary Tables**

**Supplementary Table S1 Media used in this study**

| Medium | Formula (per liter) |
| --- | --- |
| PDA | potato immersion powder 5.0 g, glucose 20.0 g, agar 15.0 g, pH7.0 |
| PSA | potato immersion powder 5.0 g, sucrose 20.0 g, agar 15.0 g, pH7.0 |
| NA | peptone 5.0 g, yeast powder 1.0 g, beef extract 3.0 g, sucrose 10.0 g, agar 15.0 g, pH7.0 |
| NB | peptone 5.0 g, yeast powder 1.0 g, beef extract 3.0 g, sucrose 10.0 g, pH7.0 |
| Protease assay  medium | glucose 15.0 g, skimmed milk powder 20.0 g, KCl 0.5 g, MgSO_4_·7H_2_O 0.5 g, agar 15.0 g, pH7.0 |
| Cellulase assay  medium | peptone 5.0 g, yeast powder 1.0 g, beef extract 3.0 g, sucrose 10.0 g, CMC-Na 5.0 g, agar 15.0 g, pH7.0 |
| Chitinase assay  medium | NH_4_H_2_PO_4_ 1.0 g, MgSO_4_·7H_2_O 0.2 g, KCl 0.2 g, colloidal chitin 10.0 g, agar 15.0 g, pH7.0 |
| β-1,3-glucanase assay medium | β-1,3-glucan 5.0 g, NaNO_3_ 2.0 g, K_2_HPO_4_ 1.0 g, KCl 0.5 g, MgSO_4_·7H_2_O 0.5 g, FeSO_4_ 0.01 g, Congo red 0.05 g, agar 15.0 g, pH7.0 |
| Chrome-azurol S (CAS) | CAS 60.5 mg, HDTMA 72.9 mg, FeCl_3_·6H_2_O 2.65 mg, NaH_2_PO_4_·2H_2_O 295.3 mg, Na_2_HPO_4_·12H_2_O 1.21 g, NH₄Cl 125 mg, KH_2_PO_4_ 37.5 mg, NaCl 62.5 mg, agar 15.0 g, pH6.8 |
| YBM | yeast extract 1.0 g, mannitol 10.0 g, K_2_HPO_4_ 0.5 g, MgSO_4_·7H_2_O 0.2 g, NaCl 0.1 g, CaCO_3_ 1.0 g, L-tryptophan 2.0 g, pH6.8 |
| ADF | Na_2_HPO_4_ 6.0 g, KH_2_PO_4_ 4.0 g, MgSO_4_·7H_2_O 0.2 g, glucose 2.0 g, gluconic acid 2.0 g, citric acid 2.0 g, FeSO_4_·7H_2_O 1.0 mg, H_3_BO_3_ 10.0 μg, MnSO_4_·H_2_O 11.19 μg, ZnSO_4_·7H_2_O 124.6 μg, CuSO_4_·5H_2_O 78.22 μg, MnO_3_ 10 μg, ACC 3.0 mM, pH 7.2 |

**Supplementary Table S2** **Specific primers for genes related to synthesis of antimicrobial peptides in *B. velezensis***

| Peptide | Gene | Primer  (5'-3') | Annealing temp  (°C) | Fragment length  （bp） |
| --- | --- | --- | --- | --- |
| Iturin | *ituA* | F: ATGAAAATTTACGGAGTATATATG | 53 | 1150 |
|  |  | R: TTATAACAGCTCTTCATACGTT |  |  |
|  | *ituD* | F: TTGAAYGTCAGYGCSCCTTT | 51 | 482 |
|  |  | R: TGCGMAAATAATGGSGTCGT |  |  |
| Bacillomycin | *bmyC* | F: AGTAAATGAACGCGCCAATC | 58 | 957 |
|  |  | R: CCCTCTCCTGCCACATAGAG |  |  |
|  | *bmyB* | F: GAATCCCGTTGTTCTCCAAA | 60 | 370 |
|  |  | R: GCGGGTATTGAATGCTTGTT |  |  |
| Surfactin | *srfAA* | F: TCGGGACAGGAAGACATCAT | 60 | 201 |
|  |  | R: CCACTCAAACGGATAATCCTGA |  |  |
| Fengycin | *fenB* | F: CTATAGTTTGTTGACGGCTC | 55 | 1400 |
|  |  | R: CAGCACTGGTTCTTGTCGCA |  |  |
|  | *fenD* | F: GGCCCGTTCTCTAAATCCAT | 60 | 269 |
|  |  | R: GTCATGCTGACGAGAGCAAA |  |  |
| Bacilysin | *bacD* | F: AAAAACAGTATTGGTYATCGCTGA | 52 | 749 |
|  |  | R: CCATGATGCCTTCKATRCTGAT |  |  |
|  | *bacA* | F: CAGCTCATGGGAATGCTTTT | 60 | 498 |
|  |  | R: CTCGGTCCTGAAGGGACAAG |  |  |

**Supplementary Table S3 Analysis of lipopeptides extracts of BR-01 by LC-MS**

| Measured mass (m/z) | Parention | Lipopeptide type |
| --- | --- | --- |
| 1008.66 | [M+H]^+^ | C_13_surfactin A or C_14_ surfactin B or C_13_surfactin C |
| 1022.67 | [M+H]^+^ | C_14_surfactin A or C_15_ surfactin B or C_14_surfactin C |
| 1036.69 | [M+H]^+^ | C_15_surfactin A or C_16_surfactin B or C_15_surfactin C |
| 1050.71 | [M+H]^+^ | C_16_surfactin A or C_16_surfactin C |
| 1044.65 | [M+H]^+^ | C_14_iturin B |
| 1058.67 | [M+H]^+^ | C_15_iturin B |
| 1072.68 | [M+H]^+^ | C_16_iturin B |
| 1086.70 | [M+H]^+^ | C_17_iturin B |
| 1100.72 | [M+H]+ | C_18_iturin B |
| 1463.79 | [M+H]^+^ | C_14_fengycin A or C_16_fengycin A or C_14_fengycin B |
| 1477.81 | [M+H]^+^ | C_15_fengycin A or C_17_fengycin A or C_15_fengycin B |
| 1491.82 | [M+H]^+^ | C_18_fengycin A or C_16_fengycin B |

**Supplementary Table S4 Experimental design of Biocontrol efficacy assays of BR-01 in greenhouse**

| Treatment  groups | Experimental operation | | Agent Application |
| --- | --- | --- | --- |
|  | Pathogens^#^ | Agent* |  |
| CK | *Xoo* or *Xoc* | NB medium | 12 h post inoculation of pathogen |
| Treatment | *Xoo* or *Xoc* | BR-01 | 12 h post inoculation of pathogen |
| Preventive | *Xoo* or *Xoc* | BR-01 | 12 h before inoculation of pathogen |
| NB medium | NB medium | NB medium | 12 h post inoculation of pathogen |

^#^The infiltration method was used for inoculation of *Xoc* and the leaf-cutting method was used for inoculation of *Xoo.* *Spraying BR-01 suspension at OD_600_ = 0.5.

**Supplementary Table S5 The antagonistic activity against rice pathogens of *Bacillus velezensis* BR-01 compared to other *B. velezensis strains***

| Strain | Antagonistic activity against rice pathogens | | | | | | References |
| --- | --- | --- | --- | --- | --- | --- | --- |
|  | *M*. oryzae | *U.* *virens* | *F.* *fujikuroi* | *R. solani* | *Xoc* | *Xoo* |  |
| BR-01 | **+** | **+** | **+** | ND | **+** | **+** | This study |
| FZB42 | **+** | - | **-** | **-** | **+** | **+** | Wu et al., 2015 |
| BC79 | **+** | **-** | **-** | **-** | **-** | **-** | Shan et al., 2013 |
| YC7007 | **+** | **-** | **+** | **-** | **-** | **+** | Chung et al., 2015 |
| G341 | **+** | **-** | **-** | **+** | **-** | **-** | Lim et al., 2017 |
| NKG-2 | **-** | **+** | **-** | **-** | **-** | **-** | Myo et al., 2019 |
| E69 | **+** | **-** | **-** | **+** | **-** | **-** | Sha et al., 2019 |
| XT1 | **+** | **-** | **-** | **-** | **-** | **-** | Torres et al., 2020 |
| 11-5 | **+** | **-** | **-** | **-** | **-** | **-** | Ma et al., 2020 |
| HN-2 | **-** | **-** | **-** | **-** | **-** | **+** | Jin et al., 2020 |
| CMRP 4490 | **-** | **-** | **-** | **+** | **-** | **-** | Teixeira et al., 2021 |
| JK23 | **-** | **-** | **+** | **-** | **-** | **-** | Zhang et al., 2021 |
| ZW10 | **+** | **-** | **-** | **-** | **-** | **-** | Chen et al., 2021 |

+, Strain with antagonistic activity against pathogens; -, no antagonistic activity was reported;

ND, the antagonistic activity was not detected.
