# Supplementary material for: Characterization of a Bacillus velezensis strain isolated from Bolbostemmatis Rhizoma displaying strong antagonistic activities against a variety of rice pathogens
Source: Front Microbiol. 2022 Sep 28;13:983781. doi: 10.3389/fmicb.2022.983781 (PMC9555170; doi:10.3389/fmicb.2022.983781)
Supplement: Supplementary file 2 [file Presentation_1.PPTX]

## Slide 1
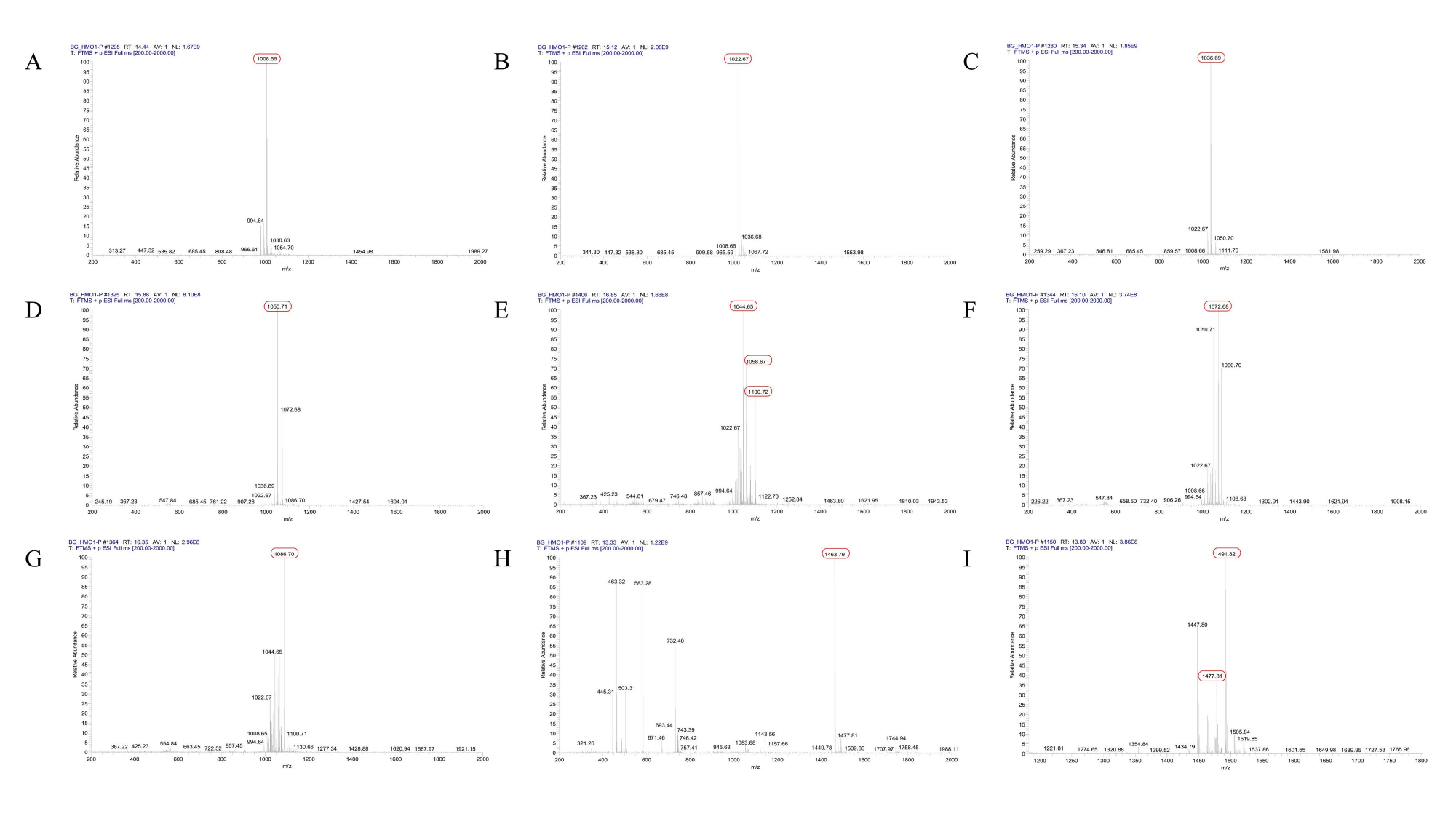

## Slide 2
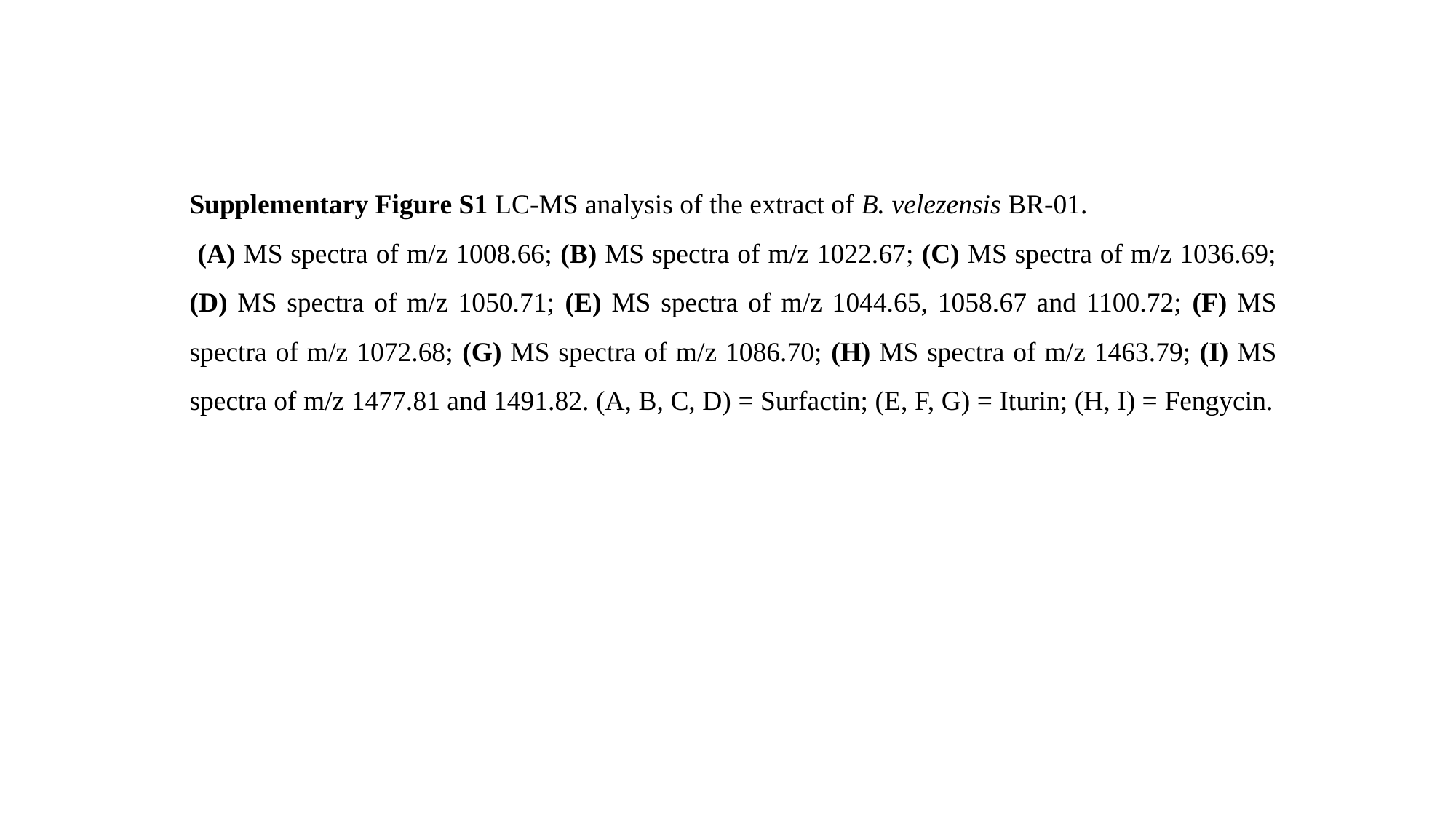

Supplementary Figure S1 LC-MS analysis of the extract of B. velezensis BR-01.
 (A) MS spectra of m/z 1008.66; (B) MS spectra of m/z 1022.67; (C) MS spectra of m/z 1036.69; (D) MS spectra of m/z 1050.71; (E) MS spectra of m/z 1044.65, 1058.67 and 1100.72; (F) MS spectra of m/z 1072.68; (G) MS spectra of m/z 1086.70; (H) MS spectra of m/z 1463.79; (I) MS spectra of m/z 1477.81 and 1491.82. (A, B, C, D) = Surfactin; (E, F, G) = Iturin; (H, I) = Fengycin.
